# Genome-wide association analysis of egg production performance in chickens across the whole laying period

**DOI:** 10.1186/s12863-019-0771-7

**Published:** 2019-08-14

**Authors:** Zhuang Liu, Ning Yang, Yiyuan Yan, Guangqi Li, Aiqiao Liu, Guiqin Wu, Congjiao Sun

**Affiliations:** 1 National Engineering Laboratory for Animal Breeding and MOA Key Laboratory of Animal Genetics and Breeding, College of Animal Science and Technology, China Agricultural University, Beijing, 100193 China; 2 Beijing Engineering Research Centre of Layer, Beijing, 101206 China

**Keywords:** Egg production, Genome-wide association study, Candidate genes, Heritability, Chicken

## Abstract

**Background:**

Egg production is the most economically-important trait in layers as it directly influences benefits of the poultry industry. To better understand the genetic architecture of egg production, we measured traits including age at first egg (AFE), weekly egg number (EN) from onset of laying eggs to 80 weeks which was divided into five stage (EN1: from onset of laying eggs to 23 weeks, EN2: from 23 to 37 weeks, EN3: from 37 to 50 weeks, EN4: from 50 to 61 weeks, EN5: from 61 to 80 weeks) based on egg production curve and total egg number across the whole laying period (Total-EN). Then we performed genome-wide association studies (GWAS) in 1078 Rhode Island Red hens using a linear mixed model**.**

**Results:**

Estimates of pedigree and SNP-based genetic parameter showed that AFE and EN1 exhibited high heritability (0.51 ± 0.09, 0.53 ± 0.08), while the h2 for EN in other stages varied from low (0.07 ± 0.04) to moderate (0.24 ± 0.07) magnitude. Subsequently, seven univariate GWAS for AFE and ENs were carried out independently, from which a total of 161 candidate SNPs located on GGA1, GGA2, GGA5, GGA6, GGA9 and GGA24 were identified. Thirteen SNP located on GGA6 were associated with AFE and an interesting gene *PRLHR* that may affect AFE through regulating oxytocin secretion in chickens. Sixteen genome-wide significant SNPs associated with EN3 were in a strong linkage disequilibrium (LD) region spanning from 117.87 Mb to 118.36 Mb on GGA1 and the most significant SNP (rs315777735) accounted for 3.57% of phenotypic variance. Genes *POLA1*, *PDK3*, *PRDX4* and *APOO* identified by annotating sixteen genome-wide significant SNPs can be considered as candidates associated with EN3. Unfortunately, our study did not find any candidate gene for the total egg number.

**Conclusions:**

Findings in our study could provide promising genes and SNP markers to improve egg production performance based on marker-assisted breeding selection, while further functional validation is still needed in other populations.

**Electronic supplementary material:**

The online version of this article (10.1186/s12863-019-0771-7) contains supplementary material, which is available to authorized users.

## Background

Egg production is the most important trait in layers as it directly affects economic benefits of poultry farmers [1]. Therefore, improving egg production is one of the main goals in a chicken breeding program. The egg production can be evaluated using different measurements, such as egg number (EN), hen-housed egg production (HHP) and egg production rate. Egg number is of great significance to select hens with higher capacity of egg laying in modern poultry breeding, especially for the country where the eggs are sold by quantity, since it can efficiently evaluate the individual egg production in a certain period. Different from EN, HHP is a good estimation of group egg production. The age at first egg (AFE) is also a very important trait for egg production as it is a partial determination of laying period.

Until now, egg production has acquired considerable improvement by the conventional selection method. However, the conventional breeding approaches could not completely eliminate the environmental effects which unavoidably results in inaccuracy of heritability estimates [2, 3]. Anang et al.(2000) reported the estimates of heritability and genetic correlation of monthly egg production in a population of White Leghorn [4]. They showed that the estimates of heritability for cumulative records were higher than monthly records, and phenotypic and genetic correlations among monthly production were high for contiguous periods. With the advances in technologies of molecular genetics and availability of single nucleotide polymorphism (SNP) markers, many studies had been performed to elucidate the genetic basis of egg production trait [5–7]. Currently, over 185 quantitative trait loci (QTL) on 24 different chromosomes (https://www.animalgenome.org/cgi-bin/QTLdb/GG/index) have been reported to be associated with age at first egg, egg number and egg production rate in chickens [8–14]. Although many QTLs were identified to exert main effect on egg production trait, some of them had wide confident intervals for position and were rarely replicated [15, 16]. A few useful QTLs can be utilized to improve breeding programs based on marker-assisted selection and best linear unbiased prediction (BLUP) [17]. A new research era began with subsequent advances in sequencing technologies and SNP chips, when genome-wide association analysis has become one of the most efficient methods to detect genetic variation in livestock. In previous researches, Liu et al. (2011), Wolc et al. (2012) and Yuan et al. (2015) reported some candidate genes for egg production, such as *ODZ2*, *GRB14*, *GTF2A1* and *CLSPN* etc. [18–22]. Most of these candidates were detected based on an F2 cross population or cross-sectional in a specific laying period, while no studies were focused on pure line population across the whole laying cycle.

In our research, we employed the commercial 600 K SNP array to identify the genomic regions and candidate genes associated with age at first egg and egg numbers in a pure line population derived from Rhode Island Red using genome-wide association study (GWAS) that could potentially accelerate the genetic improvement of egg production.

## Results

### Phenotype and genetic parameter statistics

Descriptive statistics of AFE and ENs across the whole laying period are shown in Table 1. The mean value of AFE in this population was 137 days, which meant that hens started laying eggs at about 20 weeks of age. Moreover, EN1 (egg numbers from onset of laying to 23 weeks) and EN5 (egg numbers from 61 to 80 weeks) had higher phenotypic coefficient of variation (22.67, 25.89%) than the other traits (2.27% ~ 17.27%). The pedigree-based heritability was high for AFE and EN1 (0.51 ± 0.09, 0.53 ± 0.08) and relatively low (0.09) to moderate (0.24) for EN at other stages.

**Table 1:** Descriptive statistics for AFE and egg number in different stage

*Traits*	*N*	*Mean*	*SD*	*CV(%)*	*h* ^*2*^ *(SE)*
AFE	1063	137.37d	5.7	4.15	0.51 (0.09)
EN1	1063	24.15	5.48	22.67	0.53 (0.08)
EN2	1063	95.16	2.16	2.27	0.16 (0.06)
EN3	1063	84.58	6.07	7.17	0.24 (0.07)
EN4	1060	64.71	11.17	17.27	0.23 (0.07)
EN5	1004	105.57	27.33	25.89	0.14 (0.06)
Total-EN	1063	368.13	47.84	13.00	0.09 (0.05)

*Abbreviations: AFE*: age at first egg; EN1, EN2, EN3, EN4, EN5: total egg numbers in each of five stages (from onset of laying eggs to 23 weeks, from 23 to 37 weeks, from 37 to 50 weeks, from 50 to 61 weeks and from 61 to 80 weeks); Total-EN: total egg number from onset of laying eggs to 80 weeks; *N* number of samples, *SD* standard deviation, *CV* coefficient of variance; h^2^(SE), pedigree-based heritability (standard error)

Estimates of SNP-based heritability as well as genetic and phenotypic correlations between AFE and ENs are displayed in Table 2. The pedigree-based heritability estimates were larger than those due to SNP information for all traits except for EN5 and Total-EN (0.14 vs 0.18, 0.09 vs 0.14). Genetic correlation analyses revealed that ENs in different laying period were most positively interrelated and ENs at late laying period (EN3, EN4 and EN5) had high correlation with the Total-EN.

**Table 2:** Estimates of SNP based heritabilities (on the diagonal) and of genetic (above the diagonal) and phenotypic correlations between egg production traits

*Traits*	AFE	EN1	EN2	EN3	EN4	EN5	Total-EN
AFE	**0.40 (0.05)**	–	0.14 (0.21)	−0.02 (0.15)	−0.13 (0.15)	− 0.15 (0.15)	−0.53 (0.14)
EN1	−0.95	**0.39 (0.05)**	−0.19 (0.21)	0.03 (0.15)	0.12 (0.15)	0.10 (0.15)	0.51 (0.14)
EN2	0.19	−0.19	**0.07 (0.04)**	0.64 (0.25)	0.35 (0.26)	−0.16 (0.29)	0.10 (0.29)
EN3	0.08	−0.08	0.24	**0.19 (0.05)**	0.81 (0.11)	0.38 (0.17)	0.58 (0.15)
EN4	0.03	−0.04	0.14	0.47	**0.18 (0.05)**	0.77 (0.11)	0.87 (0.07)
EN5	0.06	−0.06	0.12	0.32	0.53	**0.18 (0.05)**	0.88 (0.05)
Total-EN	−0.15	0.16	0.18	0.51	0.78	0.87	**0.14 (0.05)**

*AFE*: age at first egg; EN1, EN2, EN3, EN4, EN5: total egg numbers in each of five stages (from onset of laying eggs to 23 weeks, from 23 to 37 weeks, from 37 to 50 weeks, from 50 to 61 weeks and from 61 to 80 weeks); Total-EN: total egg number from onset of laying eggs to 80 weeks

### Genome-wide association study

**Fig. 1:**
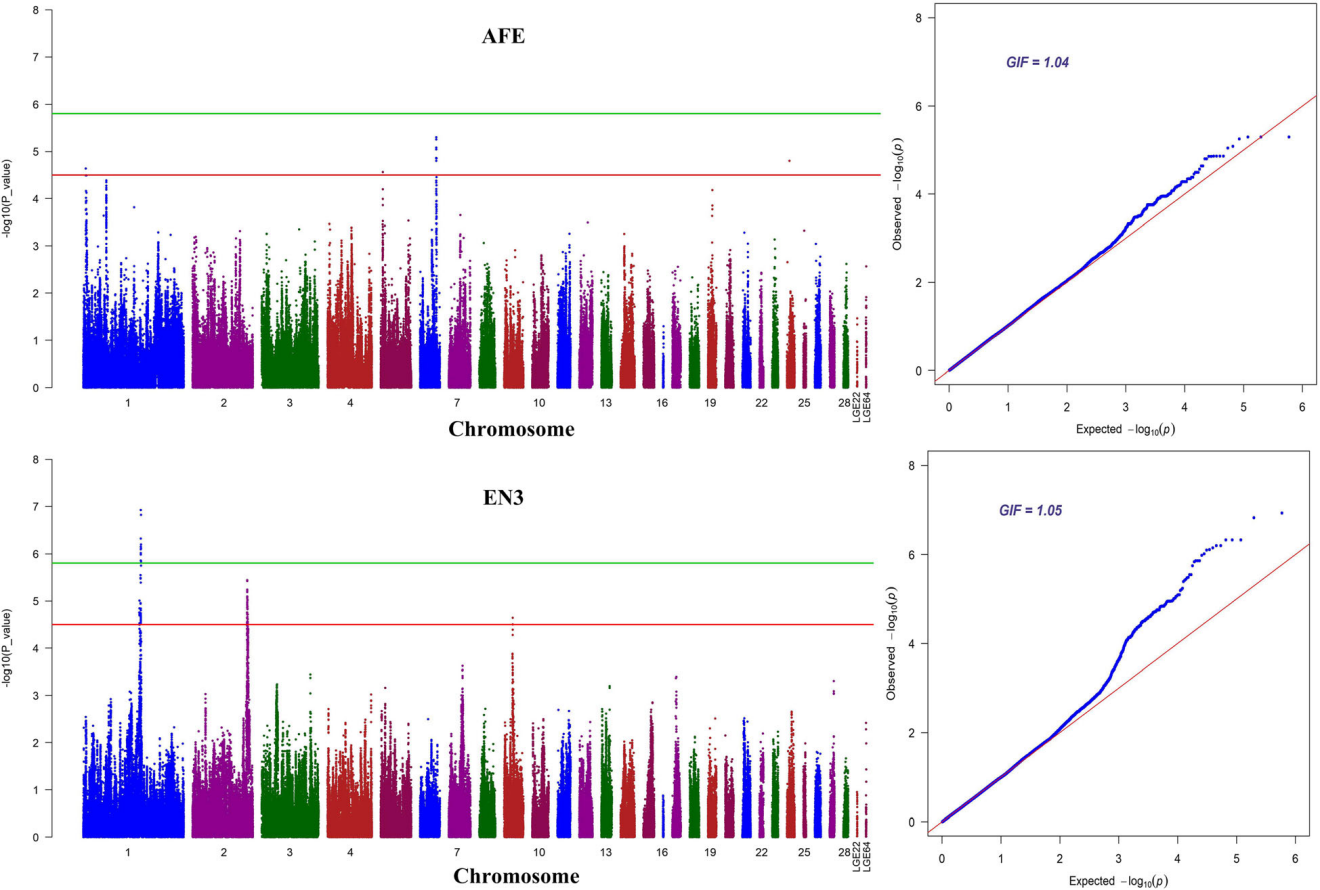
Manhattan and Q-Q plots derived from GWASs for AFE and EN3. Each dot on this figure corresponds to a SNP within the dataset, while the horizontal red and black lines denote the genome-wide significance (1.58e-6) and suggestive significance thresholds (3.17e-5), respectively. The Manhattan plot contains -log10 observed *P*-values for genome-wide SNPs (y-axis) plotted against their corresponding position on each chromosome (x-axis), while the Q-Q plot contains expected -log10-transformed *P*-values plotted against observed -log10-transformed *P*-values. GIF denotes the genomic inflation factor indicating the degree of population stratification

**Fig. 2:**
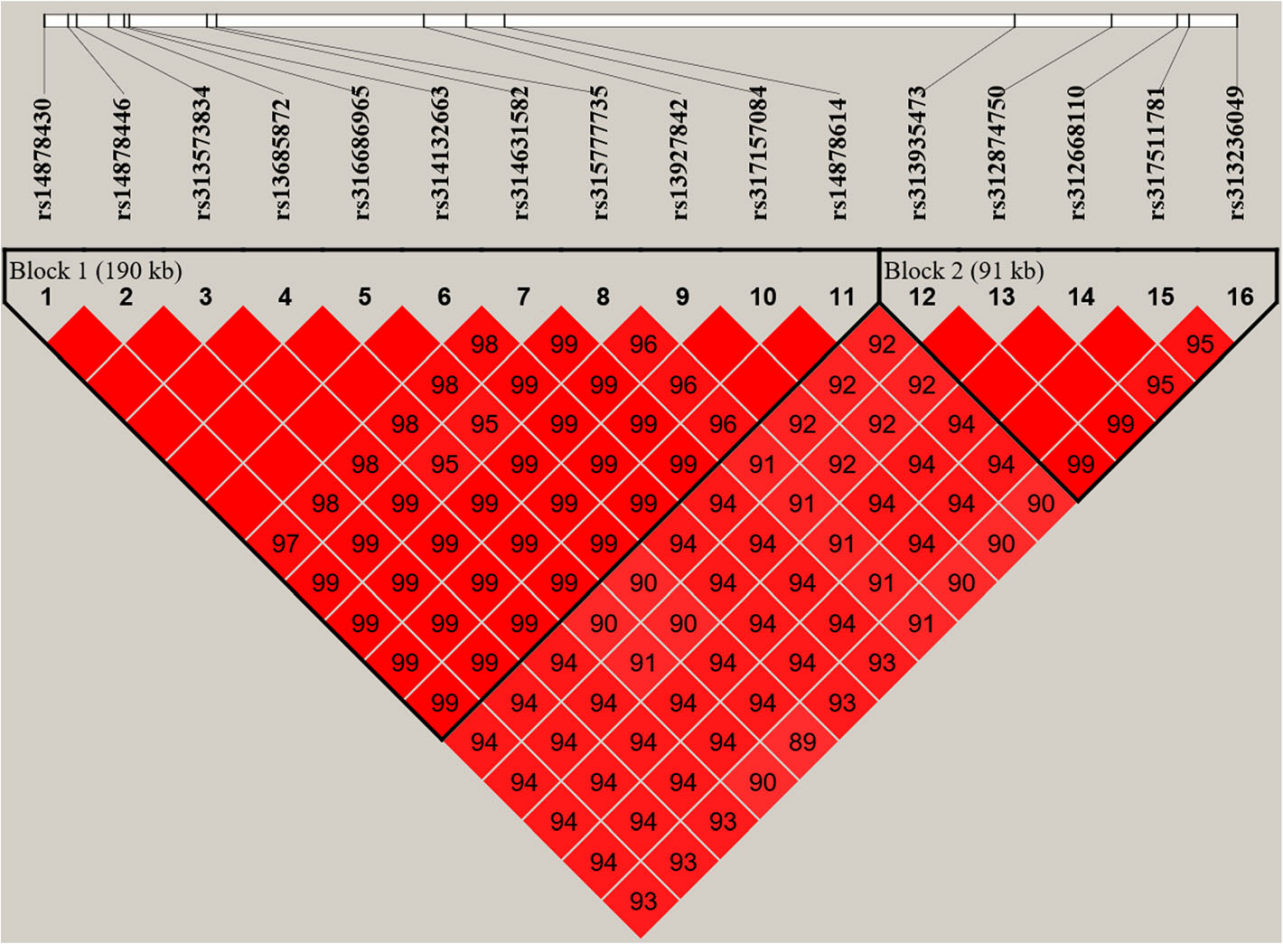
Linkage Disequilibrium (LD) analyses of SNPs in the significant region (0.49 Mb) for egg number in period 3 (EN3). LD plot of significant SNPs on GGA1 from 117.87 Mb to 118.36 Mb

Association tests for AFE and ENs were performed using a univariate linear mixed model. A total of 161 unique candidate SNPs (*P* value < 3.17E-05) located on GGA1, GGA2, GGA5, GGA6, GGA9 and GGA24 were identified (Additional file 1: Table S1). Seventeen of them scattering on four different chromosomes were suggestively associated with AFE (Table 3), while the rest containing 16 significant and 139 suggestive SNPs were related to ENs. The Manhattan and Q-Q plots for AFE and EN3 GWAS are presented in Fig. 1, which also show the genomic inflation factor (GIF) were 1.04 and 1.05, respectively. And the results of other traits are in Additional file 2: Figure. S1. For EN3, sixteen of those candidate SNPs reached genome-wide significant level (*P* value < 1.58E-06) and were located within a 0.49 Mb region that spans from 117.87 Mb to 118.36 Mb on GGA1 (Table 4). LD analysis revealed that all genome-wide significant SNPs located in above 0.49 Mb region were in strong LD status and were clustered into two blocks (Block 1: 190Kb and Block 2: 91Kb) (Fig. 2). Unfortunately, there was no genome-wide significant hits associated with EN at other stages.

**Table 3:** GWAS and annotations of suggestive SNPs (*P* value < 3.17E-05) for AFE

GGA^a^	Regions	N_SNPs^b^	Candidate/Nearst gene^c^
1	4.61 Mb ~ 4.62 Mb	2	*GATA3*
5	6,928,284	1	*HIPK3*
6	29.53 Mb ~ 29.73 Mb	13	*RAB11FIP2; FAM204A;* ***PRLHR***
24	1,994,941	1	*NTM*

^a^ Chicken chromosome; ^b^ the number of SNPs in the regions; ^c^gene name (Gallus_gallus-5.0 source)

**Table 4:** Annotation of genome-wide significant SNPs (*P* value < 1.58E-06) associated with EN3

SNP	GGA^a^	Position^b^	ALT/REF^c^	MAF^d^	Beta (SE)^e^	*P* value	Consequence	Candidate/ Nearest gene
rs14878430	1	117,871,938	C/T	0.407	0.25 (0.05)	7.96E-07	Intron	*POLA1*
rs14878446	1	117,881,774	T/C	0.408	0.25 (0.05)	1.38E-06	Intron	*POLA1*
rs313573834	1	117,885,299	A/G	0.408	0.25 (0.05)	1.38E-06	Intron	*POLA1*
rs13685872	1	117,898,459	A/G	0.417	0.25 (0.05)	1.04E-06	Intron	*POLA1*
rs316686965	1	117,904,908	T/A	0.417	0.25 (0.05)	9.65E-07	Intron	*POLA1*
rs314132663	1	117,907,038	A/G	0.408	0.25 (0.05)	1.38E-06	Intron	*POLA1*
rs314631582	1	117,939,090	C/T	0.406	0.26 (0.05)	7.05E-07	Intron	*POLA1*
rs315777735	1	117,943,070	A/G	0.418	0.27 (0.05)	1.19E-07	Intron	*POLA1*
rs13927842	1	118,028,676	C/A	0.426	0.26 (0.05)	4.70E-07	Intron	*POLA1*
rs317157084	1	118,046,119	C/T	0.426	0.26 (0.05)	4.70E-07	Downstream_43.27Kb	*PDK3*
rs14878614	1	118,062,286	C/A	0.426	0.26 (0.05)	4.70E-07	Downstream_27.35Kb	*PDK3*
rs313935473	1	118,272,993	A/G	0.423	0.24 (0.05)	1.45E-06	Upstream_3.672Kb	*APOO*
rs312874750	1	118,313,008	G/A	0.424	0.25 (0.05)	7.67E-07	Downstream_5.72Kb	*APOO*
rs312668110	1	118,340,009	G/C	0.433	0.25 (0.05)	6.31E-07	Intron	*ACOT9*
rs317511781	1	118,344,902	T/G	0.433	0.25 (0.05)	6.31E-07	Downstream_86bp	*ACOT9*
rs313236049	1	118,364,746	C/T	0.451	0.26 (0.05)	1.51E-07	Upstream_5.44Kb	*PRDX4*

^a^Chicken chromosome; ^b^Gallus_gallus-5.0 source; ^c^ALT/REF: alternative allele/reference allele; ^d^Minor allele frequency; ^e^Estimated allelic substitution effect per copy of the effect allele based on an inverse-normal transformed scale under an additive model, expressed in SD unit/allele

### Annotation of candidate SNPs

All the candidate genes associated with AFE were displayed in Table 3, including GATA binding protein 3 *(GATA3)*, homeodomain interacting protein kinase 3 *(HIPK3)*, RAB11 family interacting protein 2 *(RAB11FIP2)*, Family with sequence similarity 204 member A *(FAM204A)*, prolactin releasing hormone receptor *(PRLHR)* and Neurotrimin *(NTM)*.

The phenotypic variance of EN3 explained by the most genome-wide significant SNP (rs315777735) were estimated as 3.57% using the GCTA software. To detect promising EN3-associated genes, detailed information of sixteen genome-wide significant SNPs were annotated using the online VEP tool (Table 4). The SNP (rs315777735) was located within the intron of the DNA polymerase alpha 1, catalytic subunit *(POLA1)*, while the other SNPs were near to the candidate genes Pyruvate dehydrogenase kinase 3 *(PDK3)*, Peroxiredoxin 4 *(PRDX4)*, apolipoprotein O (*APOO*) and acyl-CoA thioesterase 9 (*ACOT9*).

## Discussion

Egg production is an important economic trait. So far, many studies have focused on the genetic determinants of AFE and EN in chicken, and most of them were based on an F2 crossed population or cross-section in a specific laying period. In earlier work, Goraga et al. (2011) and Wolc et al. (2014) reported that some QTLs on chromosomes 4, 5, 7 and 17 had an influence on EN [14, 19]. Yuan et al. (2015) subsequently carried out a GWAS in an F2 population across the whole laying period (from 21 to 72 week) [20], from which, nine candidate loci on GGA5 and two promising genes were annotated to be associated with EN. However, most of above regions or significant SNPs were population-specific and only a few candidates were found by multiple populations. In our study, we performed GWAS for AFE and EN in different stage of production using a univariate linear mixed model. This is the first GWAS using the 600 K SNP array in a Rhode Island Red pure line population across the whole laying period.

The descriptive statistics (Table 1) reveal that EN5 had the largest phenotypic variation than the other traits due to the reason that some birds died or were too old to lay eggs in this stage because of low rate of follicle development [23]. Besides, genetic parameter estimates show that AFE is a high heritable trait, which is approximately coincided with the previous reports [20, 24]. In addition, we divided the whole laying period (from onset of laying eggs to 80 weeks) into five stages according to the egg production curve and counted the total egg number in each stage, which was similar to Yuan et al. (2015). Estimate of pedigree-based heritability for EN5 was smaller than EN at the early laying stages (EN1, EN2, EN3 and EN4) probably due to the increased environmental or phenotypic variation in the late laying stage as demonstrated by Engstrom et al. [25]. Results also show that the pedigree-based heritability estimates were different with the SNP-based estimates, which were likely caused by the difference of addictive variance estimates using the pedigree or common 600 K SNPs in the animal model. Moreover, genetic and phenotypic correlations among ENs were mostly positive, especially between two neighboring stages. The egg numbers at late laying stages (EN3, EN4 and EN5) had higher correlation with the total egg number in the whole laying period than those at earlier laying stage (EN1 and EN2). These results were consistent with previous studies in White Leghorn hens [4, 26].

We carried out seven univariate genome-wide association analysis for AFE and ENs independently. Previous QTL mapping and genome-wide association studies reported some QTLs or SNPs on chromosome 1, 2, 3, 4, 5, 7, 11, 13, 20, 24 and Z were significantly associated with AFE (https://www.animalgenome.org/cgi-bin/QTLdb/GG/index) [12, 18, 19, 27]. Our GWAS for AFE did not detect any genome-wide significant SNPs, while two chromosomal regions (GGA1: 4.61 Mb ~ 4.62 Mb; GGA6: 29.53 Mb ~ 29.73 Mb) and two SNPs (located on GGA5 and GGA24) were suggestively associated to AFE. These candidate QTLs were firstly reported and six genes around these SNPs were annotated using the VEP tool, especially the Prolactin releasing hormone receptor *(PRLHR)* gene also known as *PrRPR* in chickens. Previous studies reported that *PrRP* and its structurally related peptide (C-RFa) may play distinct roles in controlling feed intake and pituitary functions in chicks [28]. In addition, *PrRP* participates in many important physiological processes to influence sexual maturity, including gonadotropin-releasing hormone, vasopressin and oxytocin secretion [29–31]. We speculate that the *PRLHR* gene has an indirect effect on AFE and further validation is required in multiple populations.

Univariate tests in egg numbers at different stages detected a total of 155 SNPs located on GGA1, GGA2, GGA6 and GGA9, only sixteen of which associated with EN3 were genome-wide significant (*P* value < 1.58E-06) and were located in a strong LD region (0.49 Mb) on GGA1 (Table 4). Furthermore, several promising genes around the significant regions were obtained. *POLA1* gene encodes the catalytic subunit of DNA polymerase alpha 1 and is an essential component of the DNA replication machinery [32, 33]. Another gene pyruvate dehydrogenase kinase isoenzyme 3 (*PDK3*) is one of the four *PDK* isoenzymes, which negatively regulates the activity of pyruvate dehydrogenase complex (*PDC*) by reversible phosphorylation [34]. The *PDK3* exhibits tissue-specific expression in testes, brain, kidney and pancreatic islets of adult [35, 36]. And the *PRDX4* is a 2-cysteine peroxiredoxin that is a major component of the endoplasmic reticulum (ER) oxidative protein folding pathway [37, 38]. Thus, *PRDX4* oxidative activity acts as a sensor to directly couple neuronal differentiation with redox environments in the ER [39]. As there are not in-depth functional researches about above genes in chickens, we speculate that they may regulate egg production via interacting with neuronal system based on studies in human. In addition, an interesting gene the apolipoprotein O (*APOO*) around the significant SNPs was also detected, which was a new member of the apolipoprotein family. Previous studies reported that *APOO* participated in fatty acid and lipid metabolism and regulated the production of lipoprotein through hormones in the chicken [40, 41]. We therefore suggest that the *APOO* is a pleiotropic gene, which affects not only the fatty deposition, but also the egg number at a specific stage in chickens. However, our study did not find a gene that affecting the egg number all the time.

## Conclusion

In conclusion, the GWAS performed in this study demonstrates that AFE is highly heritable and negatively correlated with ENs which have relatively low heritability. *PRLHR* gene may affect AFE through regulating oxytocin secretion in chickens. Moreover, four additional genes (*POLA1, PDK3*, *PRDX4* and *APOO*) identified by annotating sixteen genome-wide significant SNPs can be considered as candidates associated with EN. Findings in our research could better understand the genetic basis of egg production, while further functional validation is still needed in other populations.

## Methods

### Animals and phenotypes

A total of 1078 Rhode Island Red hens from Beijing Huadu Yukou Poultry Breeding Co., Ltd. were used in our study. The egg production and quality of this pure line has been artificially selected over ten generations. We only utilized the data from the last generation (G11) in the study, and the population was produced by crossing 92 sires and 801 dams (One sire mating 8–12 hens). Blood samples were collected from brachial veins using the standard procedure of the breeding program, which was approved by the Animal Welfare Committee of China Agricultural University.

All birds had pedigree and were housed in individual cages of the same condition. The age at first egg (AFE) and weekly egg production from onset of laying eggs to 80 weeks of age for each bird were recorded. We divided the whole laying period into 5 stages based on the egg production curve (Additional file 3: Figure. S2) and counted the egg numbers (EN) in each individual phase: the period of rapidly ascending egg production (the rate < 95% from onset of laying eggs to 23 weeks) (EN1); the period whose laying rate was over 95% was called peak of laying, which last from 23 to 37 weeks of age (EN2); while the period whose laying rate is between 90% and 95% including 37 to 50 weeks of age was set as the third phase (EN3); the fourth period with 80% to 90% laying rate was the decline phase lasting from 50 to 61 weeks (EN4); the laying rate of final phase including 61 to 80 weeks is lower than 80% (EN5). We also calculated the total egg number from onset of laying eggs to 80 weeks (Total-EN). The values used in the following analyses were derived from the rank-based inverse normal transformation of phenotypic records using the GenABEL package in R software (https://www.r-project.org/). Because the transformed data has the lower standard deviation.

### Genotyping and quality control

Genomic DNA was extracted from 1.5-mL blood samples using DNeasy 96 Blood & Tissue Kits (QIAGEN, Germany). A total of 1078 hens were genotyped with the Chicken 600 K SNP array [42] (Affymetrix, Inc. Santa Clara, CA, USA) which contained 580,961 SNPs across 28 autosomes and two sex chromosomes. We first discarded 6593 SNPs with unknown physical position and repeated genomic coordinates. The Affymetrix Power Tools v1.19.0 (APT) software was then implemented to control the quality of sample call rate (> 97%) and dish quality (> 0.82). After the quality control, 1063 individuals and 517,856 SNPs remained. In addition, the low quality of SNPs (SNP call rate < 95%, minor allele frequency < 0.01, Hardy-Weinberg equilibrium *P* < 1 × 10–6) were filtered out through the PLINK package [43]. The remaining SNPs with missing genotypes were imputed using the Beagle v4.0 procedure [44]. Finally, a total of 1063 individuals and 294,705 SNPs located on autosomes (Additional file 4: Table S2) were deemed eligible for the following analyses.

### Genome-wide association studies

Prior to the GWAS, a principal component analysis (PCA) was implemented to evaluate the population stratification using the PLINK package [43]. We pruned all SNPs to obtain independent SNPs and blocks via the option of --indep-pairwise 25 5 0.2 and –blocks-max-kb 500, respectively. A total of 14,878 independent SNPs and 16,711 linkage disequilibrium blocks were detected. The relationship matrix was built through the independent SNPs. And the principal components were calculated by the eigenvectors of the relationship matrix. Then, GWAS were carried out independently for AFE and ENs. Seven univariate association analyses which models AFE and egg numbers in different stages were performed in GEMMA software [45]. The univariate linear mixed model was as follows:

$$ \mathbf{y}=\mathbf{W}\boldsymbol{\upalpha } +\mathbf{x}\boldsymbol{\upbeta } +\mathbf{u}+\boldsymbol{\upvarepsilon} $$

In this expression, **Y** denotes the vector of phenotypic values of AFE and ENs for n samples; **W** is a matrix of covariates (including a column of 1 s and fixed effects, such as batch, top five principal components which contributing to majority of population structure variations were included in the model to control population structure); **α** is the corresponding coefficients of the fixed effects; **x** is a vector of SNP genotypes (coded as 0, 1, 2); **β** is the effect sizes of SNPs for the phenotypes; **u** is a vector of random polygenic effects with **u** ~ MVN_*n*_(0, λτ^−1^**K**) where λ represents the ratio between the two variance components and τ^−1^ denotes the variance of the residual errors, **K** is a known kinship relatedness matrix; **ε** is a matrix of errors. We applied the Wald statistical test to evaluate the null hypothesis that the SNP effect sizes for all phenotypes are zero, *H*_*0*_*:* ***β = 0*** for each SNP versus *H*_*1*_*:* ***β ≠ 0***. For each SNP in turn, GEMMA acquires either the maximum likelihood estimate or restricted maximum likelihood estimate of λ, and outputs the corresponding *P* value.

Considering the over-conservation of 5% Bonferroni correction method, we adjusted the threshold of genome-wide significant *P*-values based on the number of linkage disequilibrium blocks and the independent SNP markers [46]. Therefore, the threshold *P* values of suggestive and genome-wide significance were calculated as 3.17E-05 (1.00/31,589) and 1.58E-06 (0.05/31,589), respectively.

The Manhattan and quantile-quantile (Q-Q) plots for AFE and EN were performed using the “gap” package in the R (https://cran.r-project.org/web/packages/gap/). Genomic inflation factor (GIF) was calculated using the “GenABEL” package in the R software to judge the extent of false positive signals [47].

### Linkage disequilibrium analysis and gene annotation

We performed linkage disequilibrium (LD) analysis to further characterize causative regions associated with AFE and ENs traits by applying the solid spine algorithm in the Haploview v4.2 software [48]. Annotation of genes adjacent to candidate SNPs were obtained using the variant effect predictor (VEP) supplied by Ensembl genome browser 91(http://www.ensembl.org/Tools/VEP) [49].

### Genetic parameter and SNP effect

The pedigree-based hereditability values for AFE and ENs were estimated using a multi-trait general animal model method in the DMU v6.0 software [50]. The model was as follows:

$$ \mathrm{y}=1\upmu +\mathrm{Za}+\mathrm{e} $$

where y denotes the vector of the phenotypic values, **1** is the n × 1 vector of all 1’s,μis population means (fixed effect), **Z** is a incidence matrix of random effect (n × 1 vector), and **a** and **e** denote the additive effect and random residual error, respectively.

Moreover, the SNP-based heritability (h^2^_snp_) [51] and pairwise genetic correlations of AFE and EN were calculated using a restricted maximum likelihood (REML) approach implemented in the software GCTA v1.24 [52]. Besides, this procedure was used to estimate the phenotypic variance contribution of significant SNPs based on a genetic matrix constructed from all eligible SNPs. The models of SNP-based heritability and the phenotypic variance contribution were the same as our previously described by Yi et al. (2015) [53].

## Additional files

Additional file 1: **Table S1.** The whole information of genome-wide significant and suggestively significant SNPs for AFE and ENs using genome-wide association studies. (XLSX 21 kb)

Additional file 2: **Figure S1.** Manhattan plots for egg numbers at different stages. (PDF 537 kb)

Additional file 3: **Figure S2.** The plot of egg production curve in this population. (JPG 109 kb)

Additional file 4: **Table S2.** Basic information of SNP markers on a physical map after quality control. (XLSX 11 kb)

## Data Availability

The datasets used and/or analysed during the current study available from the corresponding author on reasonable request.

## Abbreviations

AFE: Age at first egg
EN: Egg number
GWAS: Genome-wide association study
LD: Linkage disequilibrium
QTL: Quantitative trait loci
REML: Restricted maximum likelihood
