# Supplementary material for: Genome-wide association analysis of egg production performance in chickens across the whole laying period
Source: BMC Genet. 2019 Aug 14;20:67. doi: 10.1186/s12863-019-0771-7 (PMC6693279; doi:10.1186/s12863-019-0771-7)
Supplement: Supplementary file 2 — Figure S1. Manhattan plots for egg numbers at different stages. (PDF 537 kb) [file 12863_2019_771_MOESM2_ESM.pdf]

A. Genome-wide association study for EN1 (onset to 23 weeks).

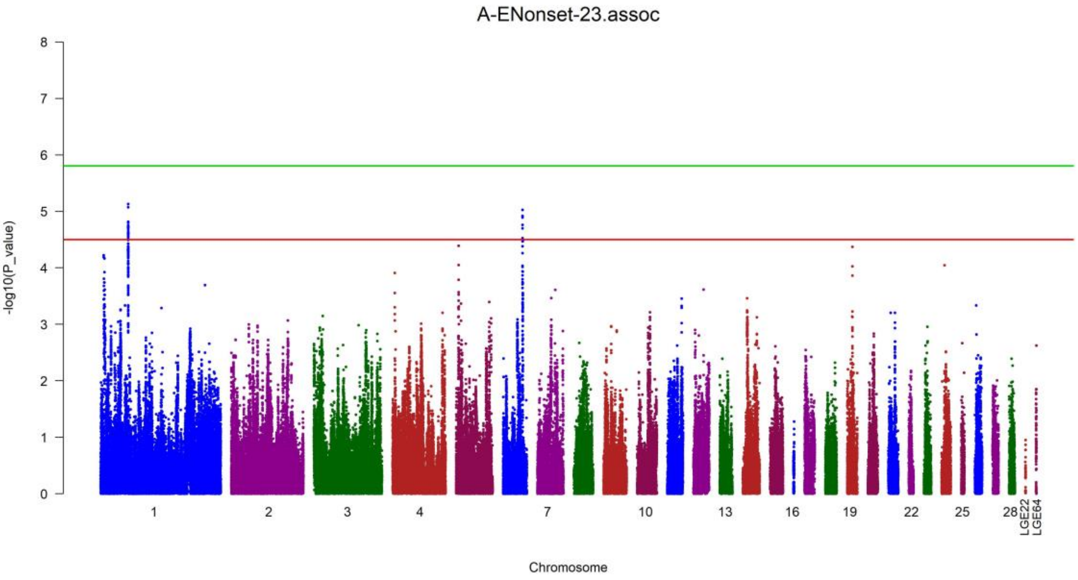

B. Genome-wide association study for EN2 (23 to 37 weeks).

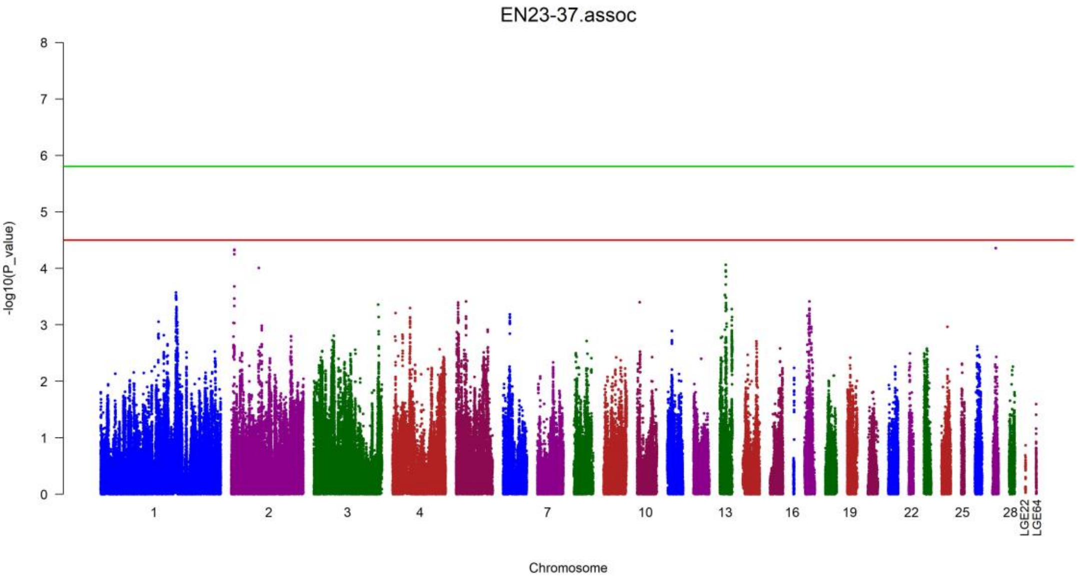

C. Genome-wide association study for EN4 (50 to 61 weeks).

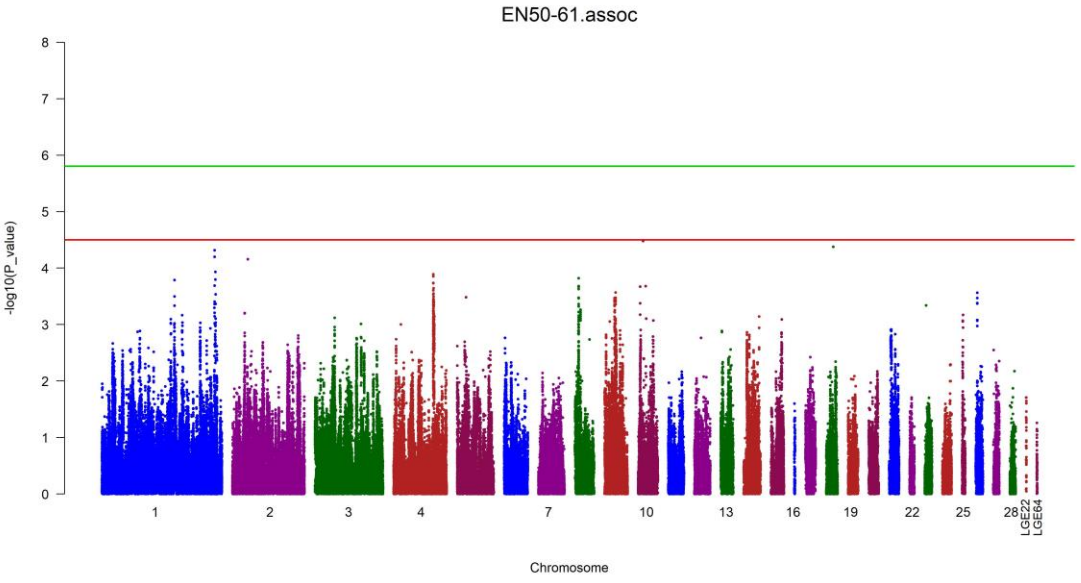

D. Genome-wide association study for EN5 (61 to 80 weeks).

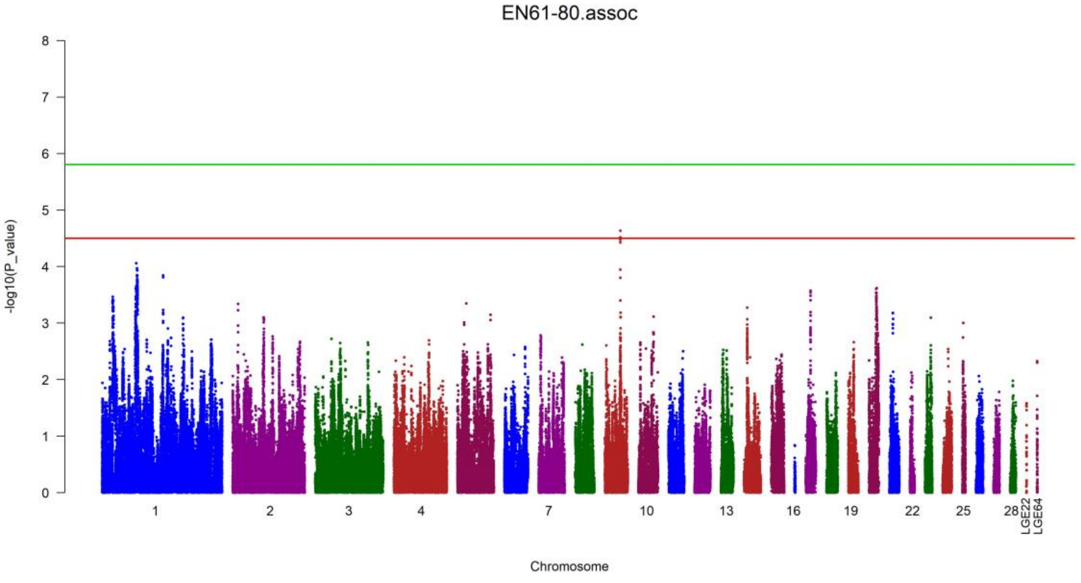

E. Genome-wide association study for Total-EN (onset to 80 weeks).

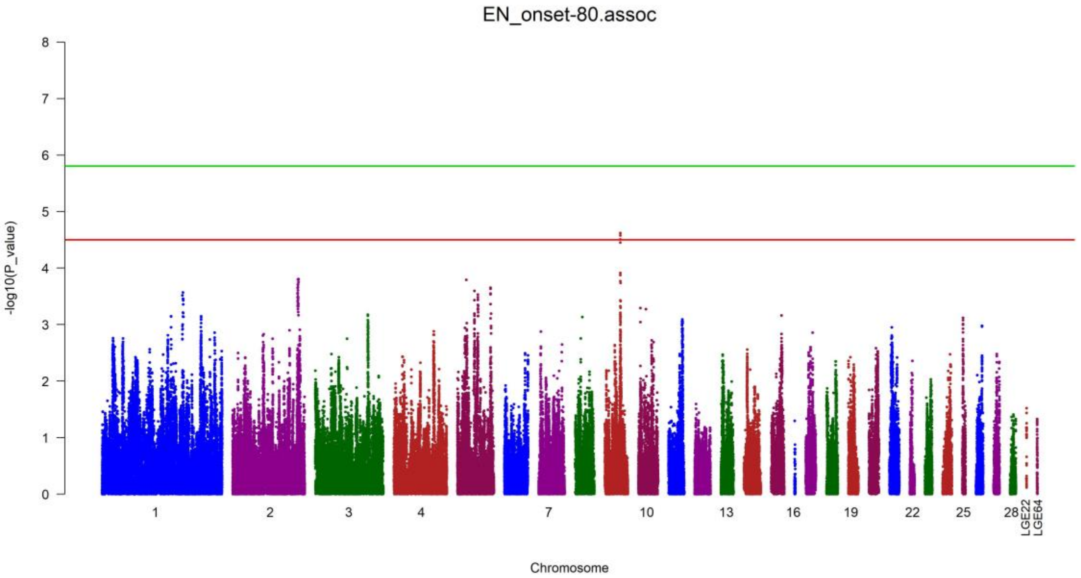

EN_onset-80.assoc
-log10(P_value)
0
1
2
3
4
5
6
7
8
1
2
3
4
7
10
13
16
19
22
25
28
LGE22
LGE64
Chromosome
