# Supplementary figures and images for: Genome-wide association analysis of egg production performance in chickens across the whole laying period
Source: BMC Genet. 2019 Aug 14;20:67. doi: 10.1186/s12863-019-0771-7 (PMC6693279; doi:10.1186/s12863-019-0771-7)

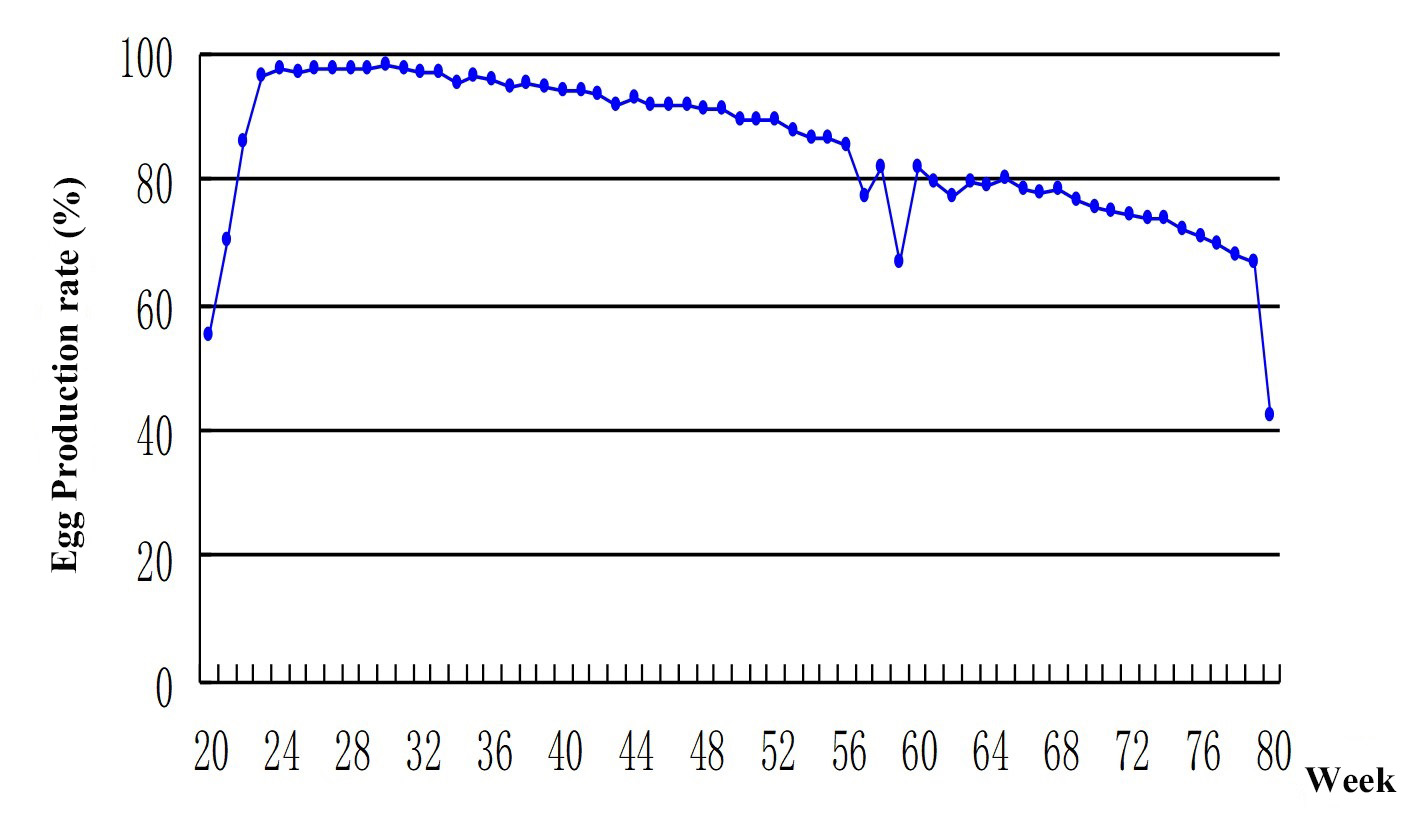

Supplement: Supplementary file 3 — Figure S2. The plot of egg production curve in this population. (JPG 109 kb) [file 12863_2019_771_MOESM3_ESM.jpg]
